# Reply to Letter to the Editor: Preoperative Albumin, Transferrin, and Total Lymphocyte Count as Risk Markers for Postoperative Complications After Total Joint Arthroplasty: A Systematic Review

**DOI:** 10.5435/JAAOSGlobal-D-20-00236

**Published:** 2021-04-22

**Authors:** Chukwuemeka Mbagwu, Matthew Sloan, Alexander L. Neuwirth, Ryan S. Charette, Keith Baldwin, Atul F. Kamath, Charles L. Nelson

*The Authors' Reply*: Thanks for the response Dr. Ives and Dr. Kuscher, you bring up an interesting point. The papers you reference show that low hand-grip strength does have clinical utility in determining patients, especially older individuals, who will likely have a longer hospital stay and/or worse outcomes, as in the stair ascent and descent.^1^ In a multicenter, retrospective, observational study of patients hospitalized with cancer, Hu et al^2^ suggest that hand-grip strength (HGS) is positively correlated with prealbumin and albumin, although it is of limited use in patients who are severely malnourished. Marcason^3^ and White^4^ mention that there is no one thing that determines malnutrition in an entire holistic sense and mentions that things to measure include HGS, muscle mass, loss of subcutaneous fat, weight loss, insufficient energy uptake, and fluid accumulation.^3,4^ Although Marcason^3^ does not agree with using albumin as a marker for malnutrition, his point that malnutrition is multivariate is something we agree with.

The focus of our paper was to delineate the clinical utility of albumin, transferrin, and total lymphocyte count in determining postoperative outcomes after total joint arthroplasty without reference to HGS. The use of albumin as a preoperative biomarker does not exclude the use of HGS as a preoperative test. It is possible that patients who are hypoalbuminemic and have a diminished hand-grip strength will have even worse outcomes than patients who are solely hypoalbuminemic or have a diminished HGS, and it is possible that either one alone is sufficient and that adding another test does not add clinical utility. The utility of why we prefer albumin over HGS is twofold: first, albumin is a laboratory value that is conveniently measured in a clinic or laboratory with any other bloodwork, and second, it is not operator-dependent with a device such as a handheld dynamometer, such as the Jamar dynamometer or Smedley dynamometer,^5^ for HGS.

Currently, no literature is available detailing the relationship between albumin, HGS, and total joint arthroplasty outcomes. This is something that may be further explored in the future.
